# Perceived usability and psychological responses to augmented reality feedback in beginner golf: a mixed-methods pilot study

**DOI:** 10.3389/fspor.2026.1938037

**Published:** 2026-09-10

**Authors:** Guan-Ren Cheng, Wen-Yu Chiu

**Affiliations:** Department of Physical Education, National Taiwan University of Sport, Taichung, Taiwan

**Keywords:** ARCS model, motor learning, pose estimation, sources of self-efficacy, technology acceptance

## Abstract

**Introduction:**

Augmented reality (AR) is increasingly used in physical education, but little is known about learners' psychological responses to AR-assisted visual feedback in beginner golf instruction.

**Methods:**

This exploratory mixed-methods pilot study examined learner perceptions and preliminary pretest–posttest changes in learning motivation and perceived sources of self-efficacy. Twenty-four undergraduates completed an eight-week multicomponent AR-supported golf program, and six participated in semi-structured interviews. Learning motivation was assessed using ARCS dimensions, and perceived sources of self-efficacy using four Bandura-based dimensions.

**Results:**

Descriptively, overall learning motivation increased by 0.31 [95% CI (0.04, 0.59)], with Cohen's *d* = 0.49 [95% CI (0.06, 0.91)]. Attention (*d* = 0.55, Holm-adjusted *p* = .052) and Relevance (*d* = 0.51, Holm-adjusted *p* = .060) showed moderate positive differences but did not reach statistical significance after adjustment. None of the four sources-of-self-efficacy dimensions reached statistical significance after Holm adjustment. Wilcoxon sensitivity analyses showed a broadly similar pattern, although Attention reached statistical significance after adjustment. Interviewed participants generally described the system as useful and easy to operate, particularly for observing, comparing, and adjusting movements.

**Discussion:**

These findings provide preliminary information about learner experiences during AR-supported golf instruction rather than evidence of effectiveness. The small sample, single-group design, multicomponent program, and absence of objective performance measures preclude causal conclusions.

## Introduction

Information and communication technologies have expanded opportunities for technology-enhanced learning (1). Augmented reality (AR) integrates virtual information with the real-world environment, supports real-time interaction, and has been applied across educational settings, including physical education and movement training (2–13). Its instructional value depends on how technological affordances are aligned with the learner, task, and instructional context (4–13).

Motor-skill learning can be challenging in sports that require precise coordination and control. In golf, novice learners often rely on demonstrations and repeated practice but may have difficulty observing their own body positions or identifying differences between their movements and an instructional demonstration. Repeated unsuccessful attempts may also influence emotional experiences and continued engagement during practice (14). Motivational and efficacy-related responses may be understood through motor-learning and self-efficacy frameworks (15, 16), while social context may also shape motor, cognitive, and affective responses during practice (17).

AR-assisted visual feedback may make movement information easier to observe and compare, although its instructional value depends on how feedback is designed and integrated into practice (10, 11, 18). Because golf-swing patterns may vary across individuals, an instructional demonstration should not be treated as a universally optimal model. Skilled golfers may use different upper-body strategies while maintaining similarly stable clubhead trajectories at ball contact (19). Accordingly, the prerecorded demonstration in this study served as a visual reference for observation and comparison rather than as a form that all learners were expected to reproduce.

This exploratory mixed-methods pilot study examined learners’ perceptions and preliminary pretest–posttest changes in learning motivation and perceived sources of self-efficacy during an eight-week AR-supported golf program. It also explored perceived usefulness, ease of use, perceived learning value, and future use. Because the study used a single-group design and did not measure objective golf performance, it was not intended to establish causal effects or compare AR-supported instruction with conventional teaching.

## Literature review

### AR-assisted feedback in physical education and motor-skill learning

AR-assisted learning has been examined in physical education and movement-based settings (4, 7, 10, 12), with its value shaped by instructional alignment (11, 18). Golf research includes real-time visual-comparison systems (21), whereas broader motor-learning work has focused mainly on performance outcomes and simplified putting tasks (20). Psychological and usability experiences during beginner golf instruction remain under explored.

### OPTIMAL theory and AR-assisted golf learning

The OPTIMAL theory identifies three conditions that can support motor learning: enhanced expectancies, autonomy support, and an external focus of attention (15). Enhanced expectancies concern positive beliefs about future performance, autonomy support provides learners with a sense of choice or control, and an external focus directs attention toward movement effects rather than body movements.

The present program offered limited connections to these principles. Learners could repeat movements, observe changes across attempts, and alternate between performing and observing, which may have supported progress expectations and some control over practice. The skeletal display, however, emphasized body positions and joint movements rather than external movement effects. The system therefore cannot be assumed to have supported all three OPTIMAL conditions. Future versions could incorporate task outcomes such as clubhead path, ball direction, or target effects.

### Learning motivation and sources of self-efficacy

The ARCS model conceptualizes motivation through Attention, Relevance, Confidence, and Satisfaction (22, 23). Real-time visualization may support Attention and Relevance, whereas Confidence and Satisfaction may depend more on successful performance. Previous AR-assisted and ARCS-based studies have reported varied motivational responses (4, 9, 24–29).

Bandura identified mastery experience, vicarious experience, verbal persuasion, and physiological and affective states as sources of self-efficacy (16). The adapted measure labels the fourth dimension Somatic and Emotional States (8), with higher scores indicating greater negative responses. These sources may reflect practice, observation, feedback, and emotional reactions but cannot be attributed specifically to AR within this multicomponent program.

### Technology acceptance and research gap

The Technology Acceptance Model emphasizes perceived usefulness and perceived ease of use (30). Semi-structured interviews examined these experiences during system use (31, 32). Because usability, motivation, and sources of self-efficacy have rarely been considered together in AR-supported beginner golf instruction, this combination formed the focus of the present exploratory study.

## Materials and methods

### Study design

This exploratory mixed-methods pilot study used a single-group pretest–posttest design supplemented by post-program semi-structured interviews. All participants took part in the same eight-week AR-supported golf program, which began on February 15, 2025. The pretest was administered before formal golf instruction and before participants were introduced to the AR-assisted system; it therefore represented their initial psychological states related to golf learning rather than a conventional-instruction baseline. The study did not include a control group, a conventional-instruction comparison condition, random allocation, or a crossover condition. Accordingly, the study was designed to examine preliminary within-group psychological changes and learner perceptions rather than to isolate the contribution of the AR component or establish causal effects.

### Participants

Twenty-four undergraduates enrolled in a university elective golf course in Taiwan participated in the study. The sample included 19 male and 5 female students aged 18–22 years. Participation was voluntary, and all participants provided written informed consent.

Eligible students had no prior formal or competitive golf training and were classified as novice learners. All 24 participants completed both the pretest and posttest questionnaires, with no withdrawals or missing questionnaire data.

After the program, six participants were selected for individual interviews using gender-stratified random sampling. Two of the five female participants and four of the 19 male participants were selected by lottery within each gender stratum. Selection was not based on questionnaire scores, learning performance, or attitudes toward the AR-assisted system. All six selected participants completed the interviews.

### Instructional procedure

The eight-week program began on February 15, 2025, and comprised 16 50-minute sessions conducted twice weekly, for a total scheduled duration of 800 min. Participants completed the learning-motivation and sources-of-self-efficacy measures before formal instruction and again after the program.

The multicomponent program included instructor explanation and demonstration, prerecorded demonstrations, individual practice, real-time skeletal visualization, and peer observation and feedback. Participants worked in pairs and alternated between performer and observer roles. The performer compared the live skeletal display with the prerecorded demonstration, received comments from the observing partner, and then repeated the movement before the roles were exchanged.

Direct AR-system exposure was estimated from the planned AR-supported practice period and the alternating pair format. Approximately 12.5–25 min per session were allocated to AR-supported practice. Because participants worked in pairs and alternated performer and observer roles, each participant was estimated to receive approximately 6.25–12.5 min of direct AR-system exposure per session, corresponding to approximately 100–200 min across the 16 sessions. Actual participant-level exposure was not electronically recorded and may therefore have varied across sessions and participants. The system supported visual comparison but did not classify movements, generate scores, or provide individualized correction.

After the final session, participants completed the posttest questionnaires. The six selected participants subsequently completed individual interviews between April 26 and May 3, 2025. Figure 4 summarizes the study procedure.

## Instruments

### AR-assisted golf learning system

#### System architecture

**Figure 1:**
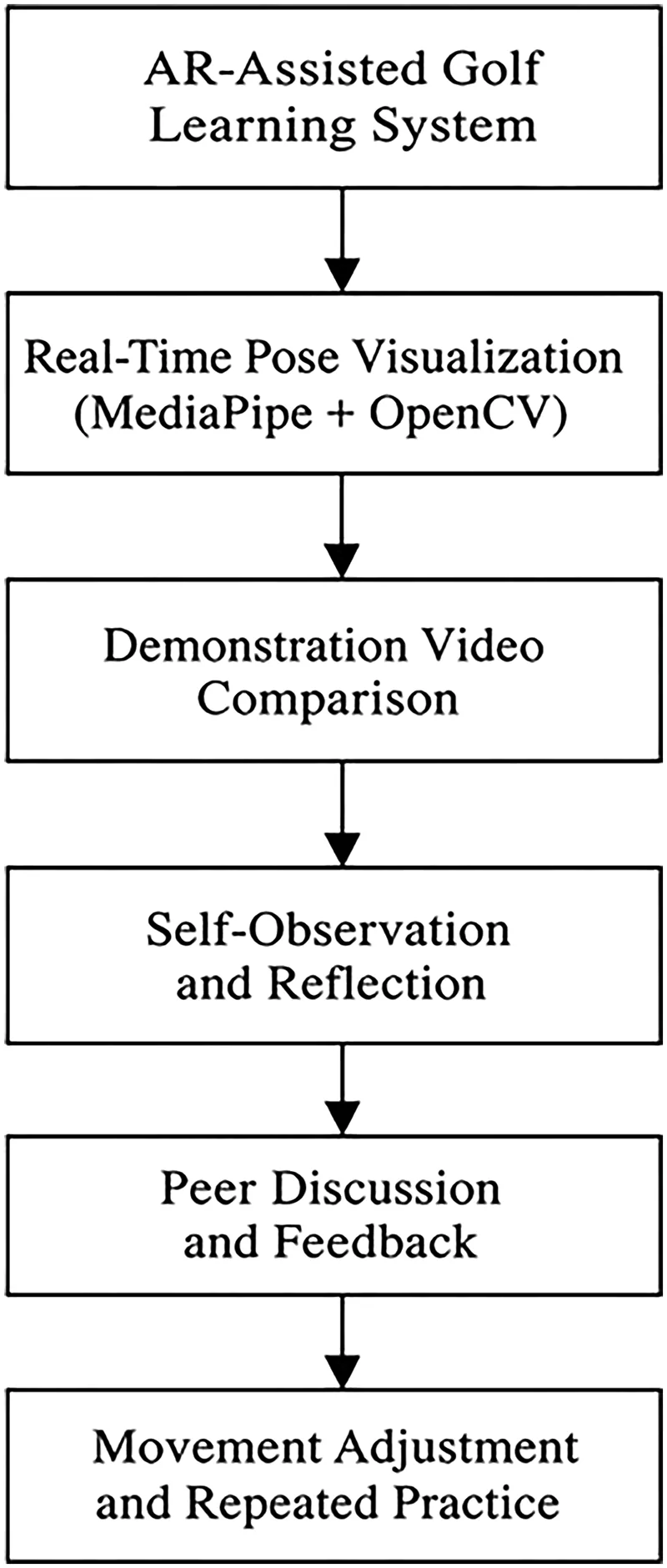
Instructional use sequence of the AR-assisted golf learning system.

The AR-assisted golf learning system used markerless pose estimation to overlay skeletal landmarks on the learner's live image and a prerecorded instructional demonstration. The system comprised three main components: image acquisition, pose estimation, and side-by-side movement comparison. A camera-equipped computer processed the live video using OpenCV and MediaPipe Pose (35) and overlaid landmarks corresponding to the head, shoulders, elbows, wrists, hips, knees, and ankles. The instructional use sequence is shown in Figure 1.

**Figure 2:**
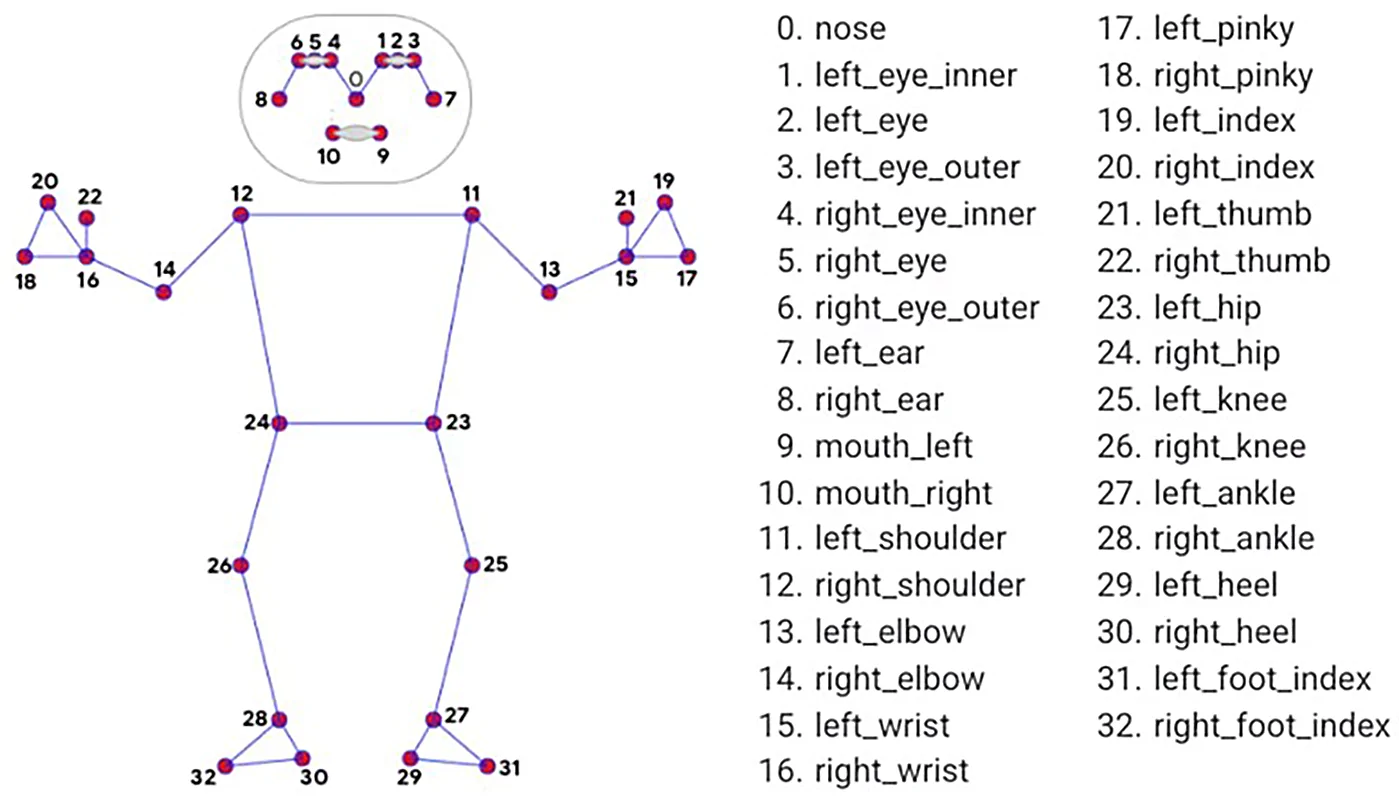
Mediapipe pose landmark indices used for real-time skeletal visualization in the AR-assisted golf learning system. Landmark labels, including the original underscore naming format, follow the source documentation. Reproduced from the google AI Edge MediaPipe Pose Landmarker documentation (35) under the CC BY 4.0 license. No changes were made to the original figure.

The prerecorded demonstration was processed using the same procedure, allowing learners to compare visible differences in body alignment, joint positions, and movement sequence. Figure 2 presents the MediaPipe Pose landmark indices used for skeletal visualization. The system did not classify movements, generate scores, or provide automated correction; instead, it served as a shared visual reference for observation and discussion.

#### Interface and functionality

**Figure 3:**
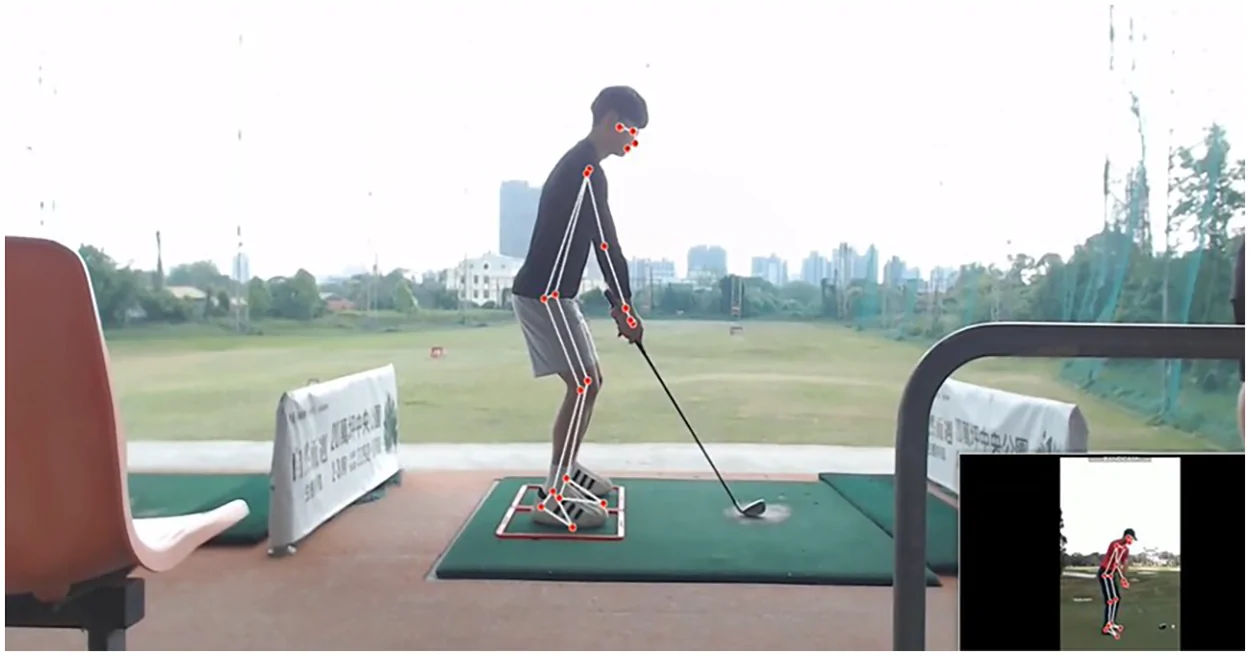
User interface of the AR-assisted golf learning system for movement observation and comparison. The facial regions were digitally blurred to protect the participant's identity.

As shown in Figure 3, the interface displayed the learner's live image and the prerecorded instructional demonstration simultaneously. During practice, learners used the skeletal displays to identify visible movement differences, discuss them with a partner, repeat the movement, and make adjustments.

**Figure 4:**
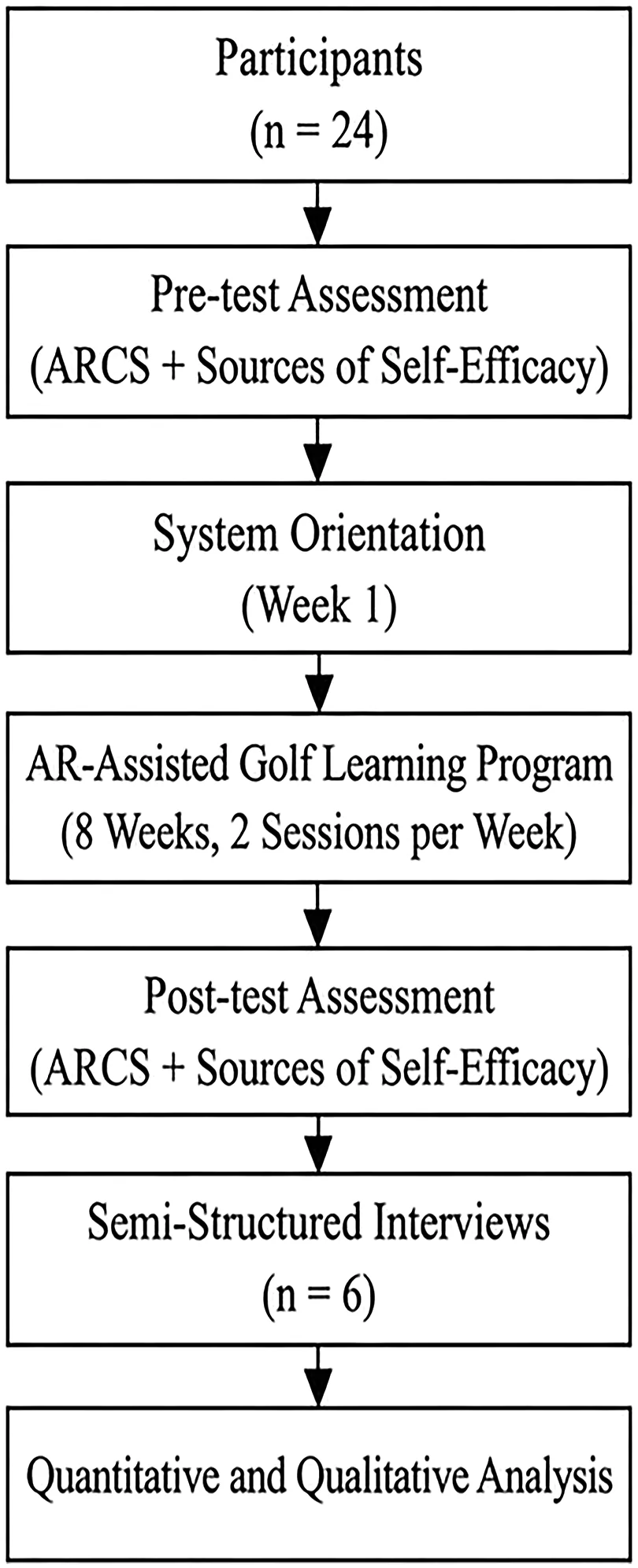
Research procedure of the multicomponent AR-supported golf instructional program.

Participants worked in pairs and alternated between performing and observing. The observing partner commented on the displayed comparison before the participants exchanged roles. The activity followed a recurring sequence of practice, observation, comparison, discussion, and adjustment.

#### Learning motivation scale

Learning motivation was measured using a 24-item ARCS-based measure adapted from Chang et al. (10). The measure included six items for each of the four dimensions: Attention, Relevance, Confidence, and Satisfaction. Items were rated on a six-point Likert scale ranging from 1 (*strongly disagree*) to 6 (*strongly agree*), with higher scores indicating greater learning motivation. In the present sample, Cronbach's *α* coefficients for the four dimensions ranged from 0.72 to 0.81.

#### Sources of self-efficacy scale

Perceived sources of self-efficacy were assessed using a 26-item scale adapted from Yu et al. (8), comprising Mastery Experience (six items), Vicarious Experience (seven items), Verbal Persuasion (six items), and Somatic and Emotional States (seven items). Items were rated from 1 (*strongly disagree*) to 6 (*strongly agree*). Higher scores indicated stronger perceptions of the first three sources, whereas higher Somatic and Emotional States scores indicated greater negative responses, such as anxiety or tension; these items were not reverse-coded. Cronbach's *α* coefficients ranged from 0.82 to 0.97. The very high coefficient for one dimension may indicate some item redundancy, particularly given the scale's adaptation from a science-education context.

Both measures were adapted to the golf-learning context. Their internal consistency was examined in the present sample, but construct validity was not independently evaluated in a movement-learning context.

### Semi-structured interviews

Individual interviews were conducted between April 26 and May 3, 2025, following the eight-week program, to explore participants’ psychological responses and learning experiences. Interviews were conducted by Guan-Ren Cheng (GC), a male master's student and research assistant trained in qualitative methods who had contributed to system development but was not involved in course instruction or grading and had no prior relationship with participants. Participants were informed of his role and the interview purpose, and all six selected participants agreed to participate.

Interviews were conducted individually at the golf course with no non-participants present, lasted approximately 30 min, and were audio-recorded and transcribed verbatim. Each participant was interviewed once; no repeat interviews or field notes were used. Interviews and coding were conducted in Chinese using the original transcripts. Quotations were translated into English by the first researcher and checked against the Chinese originals by the second researcher for accuracy and consistency. The interview guide was informed by the Technology Acceptance Model (30) and related research (31, 32), covering movement awareness, perceived learning support, motivation, confidence, perceived usefulness, ease of use, attitudes toward use, future use, and suggestions for improvement. The guide was not pilot tested. Perceived usefulness and perceived ease of use were examined qualitatively through the semi-structured interview guide; no validated usability scale was administered. Because the six-participant sample was predetermined through gender-stratified random sampling, saturation was not used as a stopping criterion.

1. Do you think augmented reality can help you learn a sport skill?
2. Did you encounter any difficulties while using the system?
3. What aspect of the course impressed you most?
4. Would you like to use augmented reality to learn sport skills in the future?
5. In what sports or learning contexts do you think this approach could be applied?
6. What suggestions do you have for improving the use of augmented reality in sport learning?

## Data analysis

### Quantitative data analysis

Quantitative data were analyzed using IBM SPSS Statistics version 27.0. Normality of paired difference scores was assessed using Shapiro–Wilk tests and Q–Q plots, and boxplots were inspected for extreme outliers. Paired-samples *t* tests examined pretest–posttest differences, with mean differences, 95% confidence intervals, and Cohen's *d* reported. Effect sizes of approximately 0.20, 0.50, and 0.80 were interpreted as small, medium, and large, respectively (34).

No *a priori* sample-size calculation was performed. With 24 paired observations, *α* = .05, and 80% power, the minimum detectable paired effect was approximately *d* = 0.60. Holm adjustments were applied separately across the four dimensions of each scale. Because separate treatment of overall learning motivation was not specified *a priori*, it was retained descriptively and not interpreted as an independently significant finding. No overall sources-of-self-efficacy score was retained, consistent with the measure's four-dimension structure. Two-sided Wilcoxon signed-rank tests were conducted as sensitivity analyses using the same adjustment structure. All analyses were exploratory.

### Qualitative data analysis

Interview data were analyzed using a hybrid deductive–inductive thematic approach. One researcher repeatedly read all six transcripts and conducted the initial coding, which was organized in Microsoft Word without dedicated qualitative-analysis software. Deductive codes reflected the research aims and technology-acceptance concepts, including perceived usefulness and ease of use, while inductive codes captured additional patterns in participants’ experiences. Related codes were compared, consolidated, and refined into 16 final codes organized under four themes: Movement Awareness and Self-Directed Adjustment, Motivational Experiences and Perceived Progress, Ease of Use and Mobile Accessibility, and Future Use and Sport-Specific Applications.

A second researcher reviewed the coding framework and preliminary themes, with differences resolved through discussion until consensus was reached. The final coding structure was checked against all six transcripts, including accounts that differed from dominant patterns. Reporting followed the Consolidated Criteria for Reporting Qualitative Research (COREQ) checklist (36); the completed checklist and a concise code-to-theme framework are provided as Supplementary Material.

### Trustworthiness

Credibility was supported through second-researcher review, comparison of codes and themes with the original Chinese-language transcripts, and participant checking. Summarized interpretations, rather than full transcripts, were returned to all six interviewees for confirmation. English quotations were checked by the second researcher against the original Chinese excerpts for accuracy and consistency. Potentially disconfirming or more qualified accounts were retained to reflect variation in participants’ perspectives. Because the interviewer was also involved in developing the system, participants may have been more inclined to provide favourable accounts of their experiences. To mitigate this risk, the second researcher independently reviewed the coding framework and thematic interpretations, helping to reduce the influence of the first researcher's expectations on the qualitative analysis. Qualitative and quantitative findings were considered together as complementary strands while preserving their distinct research questions.

## Results

### Descriptive statistics and pretest-posttest comparisons

Pretest scores represented participants’ initial psychological states before formal golf instruction, whereas posttest scores reflected their responses after the eight-week program. The analyses describe within-group changes during instruction and should not be interpreted as comparisons between conventional and AR-supported teaching. Table 1 presents descriptive statistics, mean differences with 95% confidence intervals, paired-samples *t*-test results, unadjusted and Holm-adjusted *p* values, and Cohen's *d* estimates with 95% confidence intervals. Statistical significance for the four dimensions of each measure was evaluated using Holm-adjusted *p* values. The overall learning-motivation score was retained for descriptive purposes and was not interpreted as an independently significant finding.

**Table 1:** Descriptive statistics and pretest-posttest comparisons for learning motivation and perceived sources of self-efficacy (*n* = 24).

Outcome	Pre-test *M* (SD)	Post-test *M* (SD)	Mean difference [95% CI]	*t*	Unadjusted *p*	Holm-adjusted *p*	Cohen's *d* [95% CI]
1. Learning Motivation							
Overall	4.68 (0.70)	4.99 (0.63)	0.313 [0.041, 0.585]	2.379	.026	-	0.486 [0.057, 0.905]
Attention	4.64 (0.73)	5.06 (0.71)	0.424 [0.100, 0.748]	2.709	.013	.052	0.553 [0.117, 0.978]
Relevance	4.67 (0.80)	5.06 (0.70)	0.382 [0.066, 0.698]	2.502	.020	.060	0.513 [0.080, 0.932]
Confidence	4.43 (0.82)	4.75 (0.63)	0.319 [−0.068, 0.706]	1.705	.102	.204	0.350 [−0.068, 0.757]
Satisfaction	4.98 (0.78)	5.10 (0.65)	0.125 [−0.144, 0.394]	0.963	.345	.345	0.190 [−0.210, 0.590]
2. Sources of Self-Efficacy							
Mastery Experience	4.44 (0.96)	4.68 (0.94)	0.236 [−0.105, 0.577]	1.433	.165	.660	0.292 [−0.120, 0.698]
Vicarious Experience	4.65 (0.92)	4.80 (0.88)	0.149 [−0.151, 0.449]	1.026	.316	.948	0.209 [−0.200, 0.608]
Verbal Persuasion	4.80 (0.97)	4.90 (0.96)	0.090 [−0.217, 0.397]	0.608	.549	.948	0.123 [−0.280, 0.524]
Somatic and Emotional States	3.94 (1.58)	4.26 (1.37)	0.315 [−0.395, 1.024]	0.921	.366	.948	0.188 [−0.218, 0.590]

Holm adjustments were applied separately across the four dimensions within each scale; overall scores were analyzed separately and were not included in the Holm correction families. Cohen's *d* values of approximately 0.20, 0.50, and 0.80 indicate small, medium, and large effects, respectively.

### Assumption checks

Most learning-motivation outcomes showed no substantial departure from normality. Attention showed a slight departure, *W* = .916, *p* = .047, although most observations followed the expected Q–Q pattern and no extreme outliers were identified.

For sources of self-efficacy, Vicarious Experience, *W* = .865, *p* = .004, and Verbal Persuasion, *W* = .896, *p* = .018, departed from normality. Their Q–Q plots showed upper-tail deviations, and one extreme positive difference score was identified for each outcome. Results for these dimensions were therefore interpreted with additional caution.

### Within-Group changes in learning motivation

Descriptively, overall learning motivation showed a mean pretest–posttest increase of 0.31 [95% CI (0.04, 0.59)], with an approximately moderate effect-size estimate, Cohen's *d* = 0.49 [95% CI (0.06, 0.91)]. Attention, *t*(23) = 2.709, unadjusted *p* = .013, and Relevance, *t*(23) = 2.502, unadjusted *p* = .020, also showed positive differences; however, neither reached statistical significance after Holm adjustment across the four ARCS dimensions (adjusted *p* = .052 and.060, respectively).

Effect-size estimates were approximately moderate for Attention, *d* = 0.55 [95% CI (0.12, 0.98)], and Relevance, *d* = 0.51 [95% CI (0.08, 0.93)]. Confidence and Satisfaction did not reach statistical significance after Holm adjustment, and their mean-difference and effect-size confidence intervals included zero.

### Within-group changes in sources of self-efficacy

None of the four sources-of-self-efficacy dimensions reached statistical significance after Holm adjustment. Effect-size estimates were small, and all mean-difference and effect-size confidence intervals included zero. Mastery Experience and Vicarious Experience showed small positive effect-size estimates. Somatic and Emotional States increased from 3.94 (*SD* = 1.58) at pretest to 4.26 (*SD* = 1.37) at posttest; however, this difference was not statistically significant. Because higher scores on this dimension indicate greater negative somatic and emotional responses, the numerical increase reflects a nonsignificant tendency toward greater anxiety or tension rather than improvement. The greater dispersion observed for this dimension may reflect individual differences in such responses during beginner golf learning. Results for Vicarious Experience and Verbal Persuasion were interpreted with additional caution because their paired difference scores departed from normality.

### Wilcoxon sensitivity analyses

Two-sided Wilcoxon signed-rank sensitivity analyses showed a broadly similar pattern. Overall learning motivation showed a positive difference when analyzed separately, *Z* = −2.329, *p* = .020. Attention remained statistically significant after Holm adjustment, *Z* = −2.596, adjusted *p* = .038, whereas Relevance did not, *Z* = −2.210, adjusted *p* = .081. None of the four sources-of-self-efficacy dimensions reached statistical significance after Holm adjustment. Specifically, the Wilcoxon results for Vicarious Experience and Verbal Persuasion were also nonsignificant (*Z* = −0.685, adjusted *p* = 1.000; *Z* = −0.503, adjusted *p* = 1.000, respectively), consistent with the paired-samples *t*-test findings. The complete sensitivity-analysis results are presented in Table 2.

**Table 2:** Wilcoxon signed-rank sensitivity analyses for learning motivation and sources of self-efficacy (*n* = 24).

Outcome	Wilcoxon *Z*	Unadjusted *p*	Holm-adjusted *p*
1. Learning Motivation			
Overall	−2.329	.020	-
Attention	−2.596	.009	.038
Relevance	−2.210	.027	.081
Confidence	−1.478	.139	.279
Satisfaction	−1.209	.226	.279
2. Sources of Self-Efficacy			
Mastery Experience	−1.541	.123	.494
Vicarious Experience	−0.685	.494	1.000
Verbal Persuasion	−0.503	.615	1.000
Somatic and Emotional States	−0.782	.434	1.000

Two-sided Wilcoxon signed-rank tests were used as sensitivity analyses. Holm adjustments were applied separately across the four dimensions of each scale; overall scores were analyzed separately. Holm-adjusted *p* values were calculated using the unrounded *p* values produced by SPSS. Z = standardized test statistic.

### Students’ perceptions of the AR-assisted golf learning system

The qualitative analysis generated 16 final codes organized into four themes: Movement Awareness and Self-Directed Adjustment, Motivational Experiences and Perceived Progress, Ease of Use and Mobile Accessibility, and Future Use and Sport-Specific Applications. Participants generally described the system as useful for making movement details visible, supporting comparison and adjustment, and facilitating practice. They also regarded the system as relatively easy to operate, although some indicated that its usefulness depended on the sport, the availability of appropriate devices, and the learner's familiarity with technology.

### Movement awareness and self-directed adjustment

Participants valued the side-by-side skeletal display because it made visible differences easier to notice and reduced their reliance on immediate teacher feedback.

“I can clearly see whether my movement is correct. By comparing different recordings, I can identify what needs adjustment and where I have improved.” (Participant A)

“With AR, I can discover my own movement problems instead of waiting for the teacher to point them out.” (Participant B)

These accounts indicate that participants perceived the system as a visual reference for movement awareness and possible self-directed adjustment, rather than as an automated judge of movement quality.

### Motivational experiences and perceived progress

Some participants associated visible movement information with perceived progress and continued engagement.

“The marked body points helped me understand details such as posture and joint angles. I could see my progress, which motivated me to continue learning.” (Participant B)

“When I could see that my movement was improving, I felt a sense of accomplishment and became more motivated to participate in class practice.” (Participant C)

These accounts reflect participants’ perceptions of progress and motivation and should not be interpreted as evidence of objective movement improvement.

### Ease of use and mobile accessibility

Participants generally described the system as easy to understand and operate, while also suggesting that mobile access could improve convenience and accessibility.

“The operation was not difficult at all. Overall, it was quite practical, and I could use it simply by following the instructions.” (Participant B)

“It was quite simple and straightforward: basically just start, pause, and upload.” (Participant D)

Participants also emphasized the potential convenience of accessing the system through commonly used mobile devices.

“If it could be operated through a smartphone or tablet in the future, it would be even more convenient and fit better with our daily habits.” (Participant A)

“It would be convenient. Most people have a smartphone, so if there were an app that could be opened and used directly, I would use it.” (Participant F)

These accounts indicate that participants perceived the current system as relatively easy to operate and viewed mobile-device access as a potentially more convenient mode of use. However, smartphone and tablet versions were not evaluated in the present study, so these comments reflect anticipated convenience rather than observed usability of a mobile implementation.

### Future use and sport-specific applications

Most interviewed participants expressed interest in using similar tools for unfamiliar or technically demanding skills, while also recognizing that their usefulness might depend on the sport.

“If there is a tool that helps me understand what I am doing wrong and how to improve, I would be more willing to take that course and continue using AR in the future.” (Participant A)

“Ball sports such as basketball and badminton would be suitable because movements often require specific angles and body positions. AR could help learners check whether their movements are correct and make adjustments more effectively.” (Participant A)

However, participants did not view AR-assisted feedback as equally suitable for all sports:

“It still depends on what the sport is. It may be an added benefit, but it would not necessarily be my first choice.” (Participant E)

These accounts suggest that participants perceived potential applications beyond golf, particularly for movements involving visible positional or angular features. However, they reflect anticipated usefulness rather than evidence that the approach would transfer effectively across sports.

## Discussion

Descriptively, overall learning motivation showed a positive pretest–posttest difference with an approximately moderate effect-size estimate, although the confidence interval indicated substantial uncertainty. Attention and Relevance also showed positive differences, but neither reached statistical significance after Holm adjustment across the four ARCS dimensions. Wilcoxon sensitivity analyses showed a broadly similar pattern, although Attention reached statistical significance after adjustment. None of the four sources-of-self-efficacy dimensions reached statistical significance after Holm adjustment.

Interviewed participants generally described the system as useful and easy to operate, particularly for observing and comparing movements. However, the qualitative and quantitative strands addressed different questions. The interviews described perceived usefulness, ease of use, and learning experiences, whereas the quantitative analyses examined pretest–posttest changes in learning motivation and perceived sources of self-efficacy. The interview accounts therefore provide complementary contextual information rather than confirmation of the questionnaire findings. Because AR-assisted visualization was combined with instructor guidance, demonstrations, repeated practice, and peer learning, its independent contribution cannot be determined. The findings therefore concern perceived usability and preliminary response patterns rather than causal or comparative effectiveness.

## Learning motivation

The pretest was completed before participants received formal golf instruction or used the AR-assisted system. It therefore reflected participants’ initial motivation toward golf learning rather than motivation established under conventional instruction.

Descriptively, overall learning motivation showed a positive pretest–posttest difference with an approximately moderate effect-size estimate. Attention and Relevance also showed positive differences, but neither reached statistical significance after Holm adjustment across the four ARCS dimensions. The effect-size estimates for these outcomes were approximately moderate, although their confidence intervals indicated substantial uncertainty. Wilcoxon sensitivity analyses showed a broadly similar pattern, although Attention reached statistical significance after Holm adjustment.

Real-time skeletal visualization may have made personally relevant movement information easier to notice. Participants could compare their attempts with a demonstration and relate the displayed information to their practice needs. This interpretation is consistent with previous reports of positive motivational or interest-related responses in AR-supported and ARCS-informed learning environments (4, 9, 24–28).

However, the observed pattern cannot be attributed to visualization alone. Repeated practice, increasing familiarity with golf, instructor guidance, peer interaction, system novelty, and voluntary enrollment in an elective course may also have shaped Attention and Relevance. Confidence and Satisfaction did not show statistically significant changes. Visual awareness may help learners notice movement differences without necessarily producing successful performance, a stronger sense of competence or greater satisfaction, particularly when the system does not provide scores, individualized correction, or explicit confirmation of improvement.

## Sources of self-efficacy

None of the four sources-of-self-efficacy dimensions reached statistical significance after Holm adjustment. Wilcoxon sensitivity analyses showed the same general pattern. Results for Vicarious Experience and Verbal Persuasion warrant additional caution because their paired difference scores departed from normality.

The program included experiences that could relate to Bandura's four sources of self-efficacy (16), including repeated practice, observation of instructors and peers, verbal feedback, and physical or emotional reactions during performance. These experiences, however, were not accompanied by statistically significant changes in the corresponding measures.

Small positive effect-size estimates for Mastery Experience and Vicarious Experience may reflect opportunities to repeat movements and observe demonstrations, but their confidence intervals included zero. In contrast, Somatic and Emotional States showed a numerical increase from pretest to posttest. Because higher scores on this dimension indicate greater negative somatic and emotional responses, this pattern suggests somewhat greater anxiety or tension at posttest rather than improvement; however, the change was not statistically significant. Perceived mastery should also not be interpreted as evidence of objective golf improvement because no performance outcomes were measured.

The system's feedback design may partly explain the absence of measurable changes in perceived sources of self-efficacy. It made movement differences visible but did not provide achievement indicators, individualized feedback, or explicit confirmation of successful performance. Movement awareness alone may therefore have been insufficient to alter efficacy-related interpretations during the eight-week program.

## Learners’ perceptions of system use

Interviewed participants generally described the system as useful for making movement information visible and easy to incorporate into practice. This pattern is consistent with the Technology Acceptance Model, which emphasizes perceived usefulness and perceived ease of use (30). These accounts reflect subjective experiences rather than evidence of improved movement quality or golf performance. Because only six participants were interviewed, the accounts should be treated as illustrative rather than representative.

Golf-specific evidence on real-time visual feedback remains limited. Ikeda et al. (21) developed a golf-training system that enabled comparison with an expert reference, but their study focused primarily on system development rather than learner responses or learning outcomes. More recently, Yang and Wang (12) developed an AR-assisted taekwondo coaching system that combined pose estimation with automated movement evaluation and real-time personalized feedback. Unlike that system, the present application did not classify movements, generate performance scores, or provide automated correction; instead, it served as a visual reference for observation, comparison, and discussion. In an authentic sport setting, Souissi et al. (33) reported improvements in tacking technique, execution time, and knowledge following video-supported sailing instruction. Unlike their controlled study with objective performance measures, the present single-group pilot examined perceived usability and psychological responses without assessing golf skill. Controlled studies incorporating objective performance and retention measures are therefore needed in golf.

Participants also recommended smartphone or tablet access and suggested applications in other sports. These proposals identify possible development directions, but their feasibility and instructional value require separate evaluation for each sport, movement pattern, and feedback requirement.

## Limitations and future research

The single-group pretest–posttest design did not include a control group, conventional-instruction comparison, random allocation, or crossover condition. Because the pretest represented participants’ initial psychological states before formal golf instruction, observed changes cannot be attributed specifically to the AR-assisted system. Normal learning progression, repeated practice, increasing familiarity with golf, instructor guidance, system novelty, and repeated questionnaire administration remain plausible alternative explanations.

The study also evaluated a multicomponent program rather than an AR-only intervention. Instructor demonstration, prerecorded videos, practice, real-time visualization, peer observation, feedback, and discussion were implemented together; therefore, the technological, instructional, and social contributions cannot be separated.

The sample comprised 24 students from a single elective course at one Taiwanese university, with a predominantly male composition and narrow age range. Statistical power was limited, and effects smaller than approximately *d* = 0.60 may have gone undetected. Accordingly, nonsignificant findings should not be interpreted as evidence of no change, and generalizability is limited.

Findings were based primarily on self-reported psychological measures and interviews with six participants. The construct validity of the adapted measures was not independently verified in a movement-learning context, no objective golf-performance outcomes were collected, and the interviews may not capture the full range of learner experiences. Socially desirable responses also remain possible despite separation of research activities from course grading. In addition, participant-level AR exposure was not electronically recorded, preventing precise examination of exposure–outcome relationships.

Finally, the system was developed without a formal needs assessment, focus group, or co-design process involving novice learners. Future research should use larger and more diverse samples, controlled or component-comparison designs, objective performance and retention measures, participant-level AR-usage records, and usability or co-design procedures. Longitudinal and multisite studies should also examine retention, transfer, and applicability across sports and skill levels.

## Conclusion

This mixed-methods pilot study examined learner perceptions and preliminary within-group psychological changes during an eight-week multicomponent AR-supported golf program. Overall learning motivation showed a descriptive positive pretest–posttest difference, while Attention and Relevance showed positive but nonsignificant differences after Holm adjustment. None of the four sources-of-self-efficacy dimensions reached statistical significance after adjustment.

Interviewed participants generally perceived the system as useful and easy to operate, particularly for movement observation and comparison. The side-by-side skeletal display may therefore function as a shared visual reference during beginner practice, but these perceptions should not be interpreted as evidence of objective skill improvement or instructional effectiveness.

Given the small self-selected sample, single-group multicomponent design, and absence of objective performance measures, the findings remain preliminary. Future controlled and multisite studies should include more diverse learners, objective performance and retention measures, and precise records of AR exposure.

## Data Availability

The raw data supporting the conclusions of this article will be made available by the authors, without undue reservation.
