# Supplementary material for: Perceived usability and psychological responses to augmented reality feedback in beginner golf: a mixed-methods pilot study
Source: Front Sports Act Living. 2026 Sep 10;8:1938037. doi: 10.3389/fspor.2026.1938037 (PMC13601331; doi:10.3389/fspor.2026.1938037)
Supplement: Supplementary file 1 [file Datasheet1.pdf]

Consolidated Criteria for Reporting Qualitative Research (COREQ) Checklist

Item no.	COREQ item and guide question	Reported information / location	Status
**Domain 1: Research team and reflexivity — Personal characteristics**			
1	**Interviewer/facilitator** Which author/s conducted the interview or focus group?	Guan-Ren Cheng (GC) conducted all six interviews. GC is identified as the interviewer in the revised Semi-Structured Interviews section.	Reported
2	**Credentials** What were the researcher's credentials (e.g., PhD, MD)?	At the time of data collection, GC was a master's student. This academic status is reported in the revised Semi-Structured Interviews section.	Reported
3	**Occupation** What was the researcher's occupation at the time of the study?	At the time of data collection, GC was working as a research assistant (Semi-Structured Interviews).	Reported
4	**Gender** Was the researcher male or female?	The interviewer, GC, was male (Semi-Structured Interviews).	Reported
5	**Experience and training** What experience or training did the researcher have?	GC had completed coursework in qualitative research methods and advanced research methods before conducting the interviews (Semi-Structured Interviews).	Reported
**Domain 1: Research team and reflexivity — Relationship with participants**			
6	**Relationship established** Was a relationship established prior to study commencement?	GC had no prior relationship with the six interview participants before the interviews (Semi-Structured Interviews).	Reported
7	**Participant knowledge of the interviewer** What did the participants know about the researcher (e.g., personal goals, reasons for doing the research)?	Participants were informed that GC was a member of the research team and that the interviews aimed to understand their experiences of the AR-supported course. Voluntary participation and separation from grading are also described in the Ethics Statement.	Reported
8	**Interviewer characteristics** What characteristics were reported about the interviewer/facilitator (e.g., bias, assumptions, reasons and interests in the research topic)?	GC had participated in development of the AR-assisted system but was not involved in course instruction or grading and had no prior relationship with the interviewees (Semi-Structured Interviews). Second-researcher review and examination of potentially disconfirming accounts are described under Trustworthiness.	Reported
**Domain 2: Study design — Theoretical framework**			
9	**Methodological orientation and theory** What methodological orientation was stated to underpin the study (e.g., grounded theory, discourse analysis, ethnography, phenomenology, content analysis)?	Qualitative Data Analysis reports a hybrid deductive–inductive thematic approach. Deductive coding was informed by the research aims and technology-acceptance concepts, whereas inductive coding captured additional patterns in participants' accounts.	Reported
**Domain 2: Study design — Participant selection**			
10	**Sampling** How were participants selected (e.g., purposive, convenience, consecutive, snowball)?	Participants reports gender-stratified random sampling. Two of five female participants and four of 19 male participants were selected by lottery; selection was not based on questionnaire scores, performance, or attitudes.	Reported
11	**Method of approach** How were participants approached (e.g., face to face, telephone, mail, e-mail)?	The six selected participants were approached face to face and invited to participate in the interviews (Semi-Structured Interviews).	Reported

Item no.	COREQ item and guide question	Reported information / location	Status
12	**Sample size**<br>How many participants were in the study?	Six participants completed individual interviews (Abstract; Participants; Semi-Structured Interviews).	Reported
13	**Non-participation**<br>How many people refused to participate or dropped out? Reasons?	All six selected participants agreed to be interviewed and completed the interviews; no one refused or withdrew (Participants; Semi-Structured Interviews).	Reported
**Domain 2: Study design — Setting**			
14	**Setting of data collection**<br>Where was the data collected (e.g., home, clinic, workplace)?	Individual interviews took place at the golf course immediately after the eight-week program (Semi-Structured Interviews).	Reported
15	**Presence of non-participants**<br>Was anyone else present besides the participants and researchers?	Interviews were conducted individually, one-to-one, with no non-participants present (Semi-Structured Interviews).	Reported
16	**Description of sample**<br>What are the important characteristics of the sample (e.g., demographic data, date)?	The full sample comprised 24 undergraduate novice golfers aged 18–22 years (19 male, 5 female), with no prior formal or competitive golf training. The interview sample comprised two female and four male participants selected by lottery (Participants).	Reported
**Domain 2: Study design — Data collection**			
17	**Interview guide**<br>Were questions, prompts, guides provided by the authors? Was it pilot tested?	Semi-Structured Interviews explains that the guide was informed by the Technology Acceptance Model and related research and lists six interview questions. The guide was not pilot tested.	Reported
18	**Repeat interviews**<br>Were repeat interviews carried out? If yes, how many?	Each participant was interviewed once, and no repeat interviews were conducted (Semi-Structured Interviews).	Reported
19	**Audio-/visual-recording**<br>Did the research use audio or visual recording to collect the data?	All interviews were audio-recorded and transcribed verbatim (Semi-Structured Interviews).	Reported
20	**Field notes**<br>Were field notes made during and/or after the interview or focus group?	No field notes were taken during or after the interviews (Semi-Structured Interviews).	Reported
21	**Duration**<br>What was the duration of the interviews or focus group?	Each individual interview lasted approximately 30 minutes (Semi-Structured Interviews).	Reported
22	**Data saturation**<br>Was data saturation discussed?	Data saturation was not used as a stopping criterion because the interview sample of six participants was predetermined through gender-stratified random sampling (Semi-Structured Interviews).	Reported
23	**Transcripts returned**<br>Were transcripts returned to participants for comment and/or correction?	Full interview transcripts were not returned to participants for correction. Instead, summarized interpretations were returned to all six interviewees for confirmation as participant checking (Trustworthiness).	Reported
**Domain 3: Analysis and findings — Data analysis**			
24	**Number of data coders**<br>How many data coders coded the data?	One researcher conducted the initial coding, and a second researcher reviewed the coding framework and preliminary themes; differences were resolved through discussion until consensus was reached (Qualitative Data Analysis).	Reported

Item no.	COREQ item and guide question	Reported information / location	Status
25	**Description of the coding tree** Did authors provide a description of the coding tree?	Qualitative Data Analysis reports 16 final codes organized into four themes. A concise code-to-theme framework is provided in Supplementary Table S2.	Reported
26	**Derivation of themes** Were themes identified in advance or derived from the data?	Themes were developed through a hybrid process: deductive codes reflected the research aims and technology-acceptance concepts, while inductive codes captured additional participant accounts. Related and overlapping codes were consolidated and refined into four themes (Qualitative Data Analysis).	Reported
27	**Software** What software, if applicable, was used to manage the data?	Microsoft Word was used to organize the transcripts and coding; no dedicated qualitative data-analysis software was used (Qualitative Data Analysis).	Reported
28	**Participant checking** Did participants provide feedback on the findings?	Summarized interpretations were returned to all six interviewees for confirmation (Trustworthiness).	Reported
**Domain 3: Analysis and findings — Reporting**			
29	**Quotations presented** Were participant quotations presented to illustrate the themes/findings? Was each quotation identified (e.g., participant number)?	Representative quotations are presented under the four qualitative themes and identified by participant letter (Results: Students' Perceptions of the AR-Assisted Golf Learning System).	Reported
30	**Data and findings consistent** Was there consistency between the data presented and the findings?	Interpretive statements are supported by participant quotations, and the final coding structure and themes were checked against all six complete transcripts (Qualitative Data Analysis; qualitative Results).	Reported
31	**Clarity of major themes** Were major themes clearly presented in the findings?	Four major themes are presented as subsection headings: Movement Awareness and Self-Directed Adjustment; Motivational Experiences and Perceived Progress; Ease of Use and Mobile Accessibility; and Future Use and Sport-Specific Applications (qualitative Results).	Reported
32	**Clarity of minor themes** Is there a description of diverse cases or discussion of minor themes?	Conditional and potentially disconfirming accounts are reported, including sport-specific suitability and contextual constraints such as device availability and familiarity with technology. No formal subthemes were defined (qualitative Results; Trustworthiness).	Reported

Note. COREQ = Consolidated Criteria for Reporting Qualitative Research. Checklist items and guide questions are based on Tong, Sainsbury, and Craig (2007). Locations refer to the revised manuscript and supplementary material.

Reference: Tong A, Sainsbury P, Craig J. Consolidated criteria for reporting qualitative research (COREQ): a 32-item checklist for interviews and focus groups. Int J Qual Health Care. 2007;19(6):349–357. doi:10.1093/intqhc/mzm042.
