# Supplementary material for: Perceived usability and psychological responses to augmented reality feedback in beginner golf: a mixed-methods pilot study
Source: Front Sports Act Living. 2026 Sep 10;8:1938037. doi: 10.3389/fspor.2026.1938037 (PMC13601331; doi:10.3389/fspor.2026.1938037)
Supplement: Supplementary file 2 [file Datasheet2.pdf]

Code-to-Theme Framework

This framework summarizes 16 transcript-grounded codes organized under the four themes reported in the manuscript. Participant letters indicate the interview transcripts in which each code was supported.

Final Theme	Final Code	Supporting Participants	Analytic Focus
Movement Awareness and Self-Directed Adjustment	Visual identification of movement differences or errors	A, B, C, D, E, F	Visual feedback helped participants notice movement problems that were difficult to detect during performance.
Movement Awareness and Self-Directed Adjustment	Joint-position and angle awareness	A, B, C, D, E, F	Skeletal points, lines, and replay supported awareness of joint positions, body angles, and stance.
Movement Awareness and Self-Directed Adjustment	Self-directed identification of correction needs	A, B, F	Participants described using the visual display to locate problems without relying solely on instructor explanation.
Movement Awareness and Self-Directed Adjustment	Repeated comparison and movement adjustment across attempts	A, C, E	Participants compared attempts or replayed movements and used the differences to guide subsequent adjustment.
Motivational Experiences and Perceived Progress	Perceived movement improvement or progress	A, C, F	Participants linked visual comparison and repeated practice with a sense of improvement across attempts.
Motivational Experiences and Perceived Progress	Achievement associated with improved performance	C, E, F	Improved or successful performance was associated with feelings of accomplishment.
Motivational Experiences and Perceived Progress	Increased willingness to continue the activity or course	A, B, E, F	Perceived improvement or successful performance was described as supporting continued participation.
Motivational Experiences and Perceived Progress	Perceived learning efficiency or easier skill acquisition	A, B, C	Participants described the approach as helping them learn more quickly, understand movements more clearly, or become familiar with the skill more easily.
Ease of Use and Mobile Accessibility	Simple and straightforward operation	A, B, D	Participants described the system controls and operating steps as uncomplicated.
Ease of Use and Mobile Accessibility	Perceived practicality and convenience	A, B, C, D, F	The system was generally viewed as practical or convenient for supporting practice.
Ease of Use and Mobile Accessibility	Need for assistance with computer use	E	One participant reported limited computer confidence and reliance on others for assistance, indicating variation in ease of use.
Ease of Use and Mobile Accessibility	Preference for smartphone, tablet, or app-based access	A, B, C, D, E, F	Participants anticipated greater convenience and likelihood of use if the system were available on portable devices.
Future Use and Sport-Specific Applications	Willingness to use AR in future learning	A, B, C, E, F	Participants generally expressed willingness to use the approach again, although some responses were conditional.
Future Use and Sport-Specific Applications	Applicability to other sports	A, B, C, D, E, F	Participants identified potential applications in basketball, badminton, volleyball, baseball, taekwondo, table tennis, and other sports.
Future Use and Sport-Specific Applications	Suitability depends on sport or task characteristics	A, E	Participants noted that the value of AR may vary by sport, with some activities viewed as less suitable than others.
Future Use and Sport-Specific Applications	Fine-grained analysis of sport-specific movements	A, B, C, D, E, F	Participants emphasized potential value for examining technical details such as shooting angles, swing mechanics, stance, joint positions, and other precise movement features.

Note. The framework is intended as a concise audit trail linking the coded interview content to the four themes. It does not imply that every code was expressed by every participant.
